# Evaluation of GeneXpert PA assay compared to genomic and (semi-)quantitative culture methods for direct detection of *Pseudomonas aeruginosa* in endotracheal aspirates

**DOI:** 10.1186/s13756-021-00978-9

**Published:** 2021-07-23

**Authors:** Thomas Ewout van der Schalk, Jasmine Coppens, Leen Timbermont, Agata Turlej-Rogacka, Liesbet Van Heirstraeten, Matilda Berkell, Li Yu, Christine Lammens, Basil Britto Xavier, Veerle Matheeussen, Margareta Ieven, Michael McCarthy, Philippe G. Jorens, Alexey Ruzin, Mark T. Esser, Samir Kumar-Singh, Herman Goossens, Surbhi Malhotra-Kumar

**Affiliations:** 1 Laboratory of Medical Microbiology, Vaccine and Infectious Disease Institute , University of Antwerp, Wilrijk, Belgium; 2 Laboratory of Clinical Microbiology, Antwerp University Hospital , Edegem, Belgium; 3 Microbial Sciences, BioPharmaceuticals R&D, AstraZeneca, Gaithersburg, USA; 4 Statistical Sciences, BioPharmaceuticals R&D, AstraZeneca, Gaithersburg, USA; 5 Early-Stage Development, Cardiovascular, Renal and Metabolism, BioPharmaceuticals R&D, AstraZeneca, Gaithersburg, USA; 6 Department of Intensive Care Medicine , Antwerp University Hospital , Edegem , Belgium; 7 Molecular Pathology Group, Cell Biology and Histology, University of Antwerp, Wilrijk, Belgium

**Keywords:** Cepheid, GeneXpert, Real-time PCR, Rapid diagnostics, VAP, Ventilator-associated pneumonia, ChromID^®^*P. aeruginosa*, Chromogenic medium, *Pseudomonas aeruginosa*

## Abstract

**Introduction:**

*Pseudomonas aeruginosa* is a common cause of ventilator-associated pneumonia (VAP). Rapid and accurate detection of lower respiratory tract colonization and/or infection with *P. aeruginosa* may advise targeted preventive (antibody-based) strategies and antibiotic therapy. To investigate this, we compared semi-quantitative culture results from 80 endotracheal aspirates (ETA) collected from mechanically-ventilated patients, to two culture and two non-culture-based methods for detection of *P. aeruginosa.*

**Methods:**

*P. aeruginosa*-positive (n = 40) and -negative (n = 40) ETAs from mechanically ventilated patients analyzed initally by (i) routine semi-quantitative culture, were further analyzed with (ii) quantitative culture on chromogenic ChromID *P. aeruginosa* and blood agar; (iii) enrichment in brain heart infusion broth followed by plating on blood agar and ChromID *P. aeruginosa*; (iv) O-antigen acetylase gene-based TaqMan qPCR; and (v) GeneXpert PA PCR assay.

**Results:**

Of the 80 ETA samples included, one sample that was negative for *P. aeruginosa* by semi-quantitative culture was found to be positive by the other four methods, and was included in an “extended” gold standard panel. Based on this extended gold standard, both semi-quantitative culture and the GeneXpert PA assay showed 97.6% sensitivity and 100% specificity. The quantitative culture, enrichment culture and O-antigen acetylase gene-based TaqMan qPCR had a sensitivity of 97.6%, 89.5%, 92.7%, and a specificity of 97.4%, 100%, and 71.1%, respectively.

**Conclusion:**

This first evaluation of the GeneXpert PA assay with ETA samples found it to be as sensitive and specific as the routine, hospital-based semi-quantitative culture method. Additionally, the GeneXpert PA assay is easy to perform (hands-on time ≈ 5 min) and rapid (≈ 55 min assay time). The combination of the high sensitivity and high specificity together with the rapid acquisition of results makes the GeneXpert PA assay a highly recommended screening technique. Where this equipment is not available, semi-quantitative culture remains the most sensitive of the culture methods evaluated here for *P. aeruginosa* detection in ETA samples.

**Supplementary Information:**

The online version contains supplementary material available at 10.1186/s13756-021-00978-9.

## Background

*Pseudomonas aeruginosa* infections primarily affect immunocompromised patients and this opportunistic pathogen frequently harbours multiple antibiotic resistance mechanisms [1]. *P. aeruginosa* is also a frequently-occurring nosocomial pathogen causing life-threatening infections such as ventilator-associated pneumonia (VAP) in the critically ill patient necessitating ventilation [2]. VAP occurs after endotracheal intubation and is estimated to affect up to 30% of mechanically ventilated patients [3]. In addition to increasing patient morbidity and mortality, VAP is associated with prolonged hospital stay and increased healthcare costs, and is classically detected based on clinical signs, a new infiltrate on chest X-rays and, importantly, detection of the causative pathogen in respiratory samples [3, 4].

Bacterial colonization is a well-known risk factor for VAP [5]. Therefore, identification of colonizing pathogens by surveillance cultures is employed as part of a pre-emptive strategy for VAP. Because endotracheal aspirates (ETAs) can be acquired easily from intubated patients with limited complications, collection of ETAs represents a relatively non-invasive procedure and these samples are commonly used for surveillance microbiological cultures [6].

While culture of pathogens remains the gold standard, molecular tests that typically have a shorter turnaround time can drastically decrease the critical time-to-initiation of preventive and therapeutic strategies, including the initiation of the appropriate antibacterial treatment. In this study, we evaluated the current research use only GeneXpert PA assay, which directly detects *P. aeruginosa* from ETA samples, and compared it to culture and non-culture based methods for detection of *P. aeruginosa* in ETA samples.

## Materials and methods

### Study design and sample collection

**Fig. 1:**
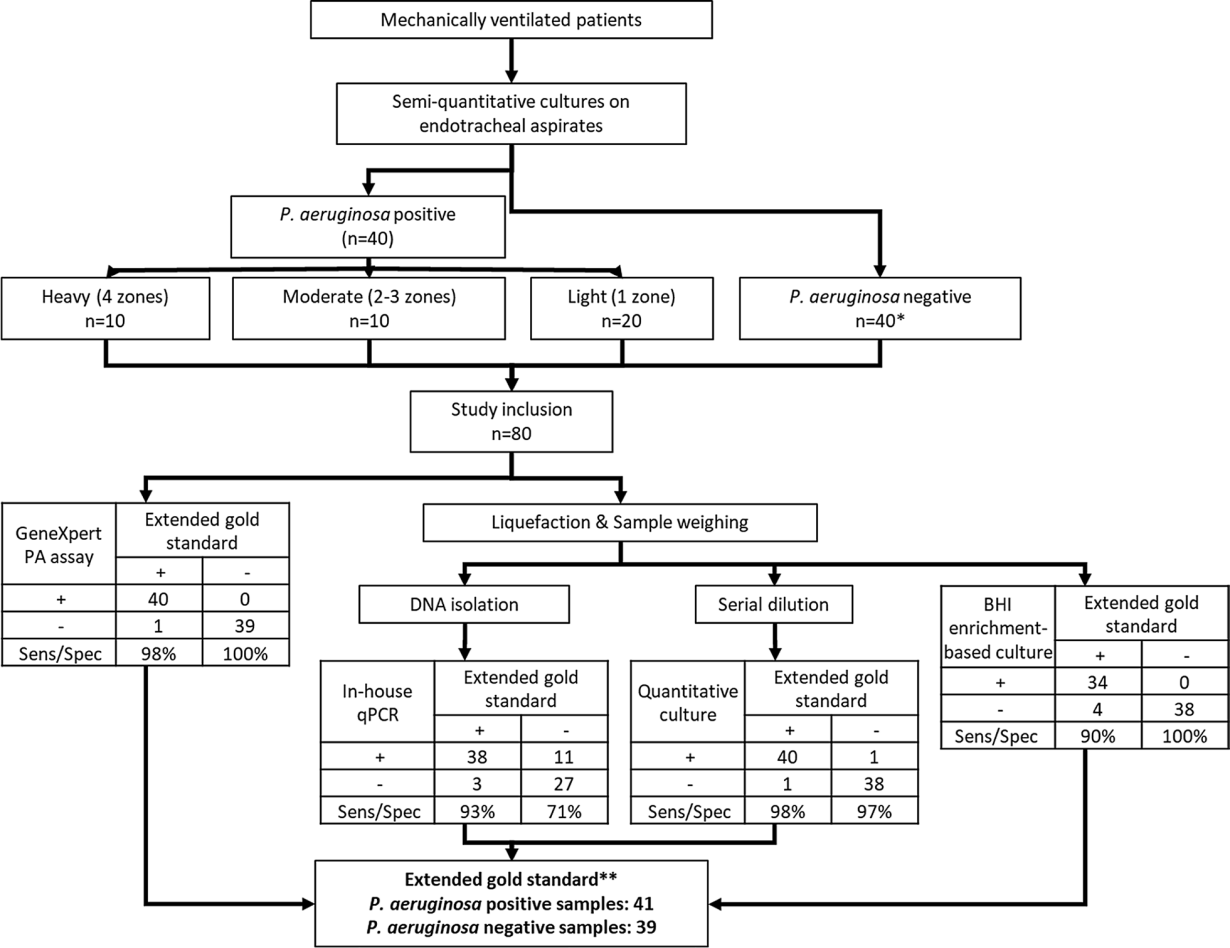
Study flowchart showing the ETA sample selection and processing order within the study. *Negative samples were included consecutively every time a positive sample was included. **Sample 70 (Additional file 1: Table S1) was found negative in semi-quantitative culture but positive in all other assays resulting in the extended gold standard. *Sens* sensitivity, *Spec* specificity

The study was designed to assess the performance of a rapid screening test, the Cepheid GeneXpert PA assay, in patients at risk of developing VAP. During February 2017–September 2018, ETA samples were collected from mechanically ventilated adult patients admitted to the intensive care unit (ICU) at the Antwerp University Hospital (UZA), either for surveillance cultures or as routine samples obtained in patients with a suspected (pulmonary) infection (Fig. 1). Clinical patient data regarding the development of VAP was also collected in the patient data management system (Metavision, iMD soft). VAP was diagnosed by a board certified senior ICU physician according to the most stringent and “validated” definition: a new bacterial pneumonia present in patients receiving mechanical ventilation for at least 48 h as characterised by a new infiltrate on the chest radiograph, signs of infection and detection of a bacterial causative agent [7], the latter most using semi-quantitative culture results as part of routine clinical practice. ETAs were analysed immediately at the UZA Clinical Microbiology Laboratory using quadrant-based, semi-quantitative culture on blood agar, chocolate agar, McConkey, and colistin-nalidixic acid agar (CNA, Oxoid, the UK) where bacterial growth was evaluated after 24 h of incubation [8]. Sample inclusion in the study was based on semi-quantitative culture results from the UZA Clinical Microbiology Laboratory for routine diagnosis of VAP. Based on the quadrant growth, the semi-quantitative culture method categorizes positive samples as light (growth in quadrant one), moderate (growth in quadrants two and three) and heavy (growth in all four quadrants). If positive, the sample was included in this study with the corresponding classification (Fig. 1). Following a *P. aeruginosa*-positive sample, the first subsequent semi-quantitative culture-negative sample was included in the negative group (Fig. 1). At the end of the study period, 80 samples were collected consisting of 40 each where *P. aeruginosa* was either detected or not. Among the *P. aeruginosa*-positive samples, 20, 10, and 10 were classified as light, moderate, and heavy *P. aeruginosa* loads based on semi-quantitative culture, respectively (Fig. 1, Additional file 1: Table S1). Collected ETA samples were stored at 4 °C for a maximum of 48 h. Upon inclusion in the study, they were further processed with different methods for *P. aeruginosa* detection and quantitation at the Laboratory of Medical Microbiology, University of Antwerp. Methods used were: GeneXpert PA assay; quantitative culture on chromogenic ChromID^®^ *P. aeruginosa* and blood agar; brain heart infusion (BHI) broth enrichment followed by blood agar and ChromID^®^ *P. aeruginosa* plating; and quantitative TaqMan real-time PCR (qPCR) targeting the O-antigen acetylase on extracted DNA. The study was approved by the UZA ethics committee (Belgian registration number B300201629199).

### GeneXpert^®^ PA assay

The GeneXpert^®^ PA assay (Cepheid, USA) is a PCR test that is currently available for research use. The assay was performed according to the manufacturer's instructions. Briefly, ETA samples were adsorbed onto a Cepheid collection device swab (CLASSIQ, COPAN), dissolved in elution buffer and vortexed at high speed for 10 s (Scientific Industries Inc., USA). From the elution reagent, 1.7 ml was transferred to the cartridge and analysed by GeneXpert^®^ Dx system v4.7b (Cepheid, USA). The overall process of extraction, amplification, and detection of intact bacterial cells was completed in 55 min. Samples were reported by the accompanied GeneXpert software as either *P. aeruginosa* “detected” or “not detected”. The detection was based on the *P. aeruginosa* PCR cycle threshold (Ct) values and the Ct values of the sample adequacy control (a multiplexed control that contains primers and probes for the detection of human cells or DNA and should only be considerd in case of a *P. aeruginosa*-negative result) and sample processing control (a control containing *Bacillus globigii* spores for verification of successful bacterial cell lysis) with valid Ct ranges between 3 and 45 for all three targets.

### Sample preparation for comparator culture-based and qPCR methods

After initiating the GeneXpert assay, the remaining ETA sample was homogenised by blending and liquefaction. All samples were blended with the dispersing instrument T10 basic ULTRA-TURRAX (IKA, Staufen, Germany) for 10 s at maximum speed on ice. Depending on the viscosity of the sample, checked by visual inspection, the blending time was increased by steps of 10 s, with a maximum blending time of 60 s. After blending, the samples were liquefied with lysomucil (10% *N*-acetylcysteine, Zambon, Milan, Italy). Three ml lysomucil was dissolved in 12 ml phosphate-buffered saline, and an equal amount in volume (≈ 300 µl) of liquefying reagent was added to the sample, and vortexed at full speed for 10 s. After incubation at 37 °C for 15 min, the samples were vortexed again at full speed for 10 s, and the incubation step was repeated. The sample was then split up in ≈ 400 µl for quantitative culture and enrichment-based culture, and ≈ 200 µl for O-antigen acetylase gene-based qRT-PCR.

### Quantitative cultures

Serial dilutions of the liquefied samples were spirally-plated (Eddy Jet, program 6; 50 µl logarithmic spreading; IUL, Spain) on chromogenic ChromID^®^ *P. aeruginosa* medium (bioMerieux, France) and on blood agar (Columbia II Agar Base, Oxoid) with 5% defibrinated horse blood. After 24 h of incubation, violet to orange *P. aeruginosa* colonies on the ChromID^®^ *P. aeruginosa* were counted and when no growth of *P. aeruginosa* was detected, the blood agar plate was screened for presence of *P. aeruginosa*. At least one presumptive *P. aeruginosa* colony per sample was speciated by MALDI-TOF (Bruker, USA) and *P. aeruginosa* loads were calculated as colony-forming units (CFU)/ml for each sample. Limit of detection was 40 CFU/ml with this method on both blood and ChromID^®^ *P. aeruginosa* agar.

### Enrichment-based cultures

Additional enrichment was performed by overnight incubation of a small leftover volume (≈ 100 µl) of the liquefied sample in BHI broth followed by plating on ChromID^®^ *P. aeruginosa* as well as on blood agar plates. Presumptive *P. aeruginosa* colonies were confirmed by MALDI-TOF (Bruker, USA).

### O-antigen acetylase gene-based qRT-PCR

200 µl of the liquefied sample was subjected to proteinase K treatment for 15 min at 56 °C followed by automated DNA extraction (NucliSENS^®^ EasyMag^®^, bioMérieux SA, France) and frozen until batch analysis by an in-house developed qPCR assay. Concentrations of *P. aeruginosa* DNA in samples were determined using qPCR targeting the O-antigen acetylase gene using previously designed primers [9] in combination with an in-house developed Taqman probe. The assay was performed in a 20 µl reaction volume containing 10 µl of 2x SensiFAST™ Probe No-ROX Kit (Bioline, London, UK), 400 nM concentrations of primers PA431CF (CTGGGTCGAAAGGTGGTTGTTATC) and PA431CR (GCGGCTGGTGCGGCTGAGTC), and 150 nM TaqMan probe (cy5-CGAACAGCGCATTCACGTAGG-BBQ) together with 4 µl of DNA template. Amplification was carried out on the CFX96 Touch™ Real-Time PCR detection system (Bio-Rad Laboratories Inc, California, USA) using the following cycling parameters: 5 min at 95 °C and 40 cycles of 10 s at 95 °C and 50 s at 60 °C. Bacterial loads were calculated based on a standard curve that was set up using Avogadro’s constant and the molecular weight of serially diluted O-antigen acetylase gene PCR product from *P. aeruginosa* ATCC27853 [10]. Samples and standard curves were run in triplicate and were considered valid when at least two out of the three replicates had a Ct with less than 0.2 difference. The qPCR assay was validated with *P. aeruginosa-*negative ETAs spiked with different concentrations of *P. aeruginosa* before utilization in this study (data not shown).

### Statistical analysis

Normality was assessed using Shapiro’s test followed by statistical comparisons of patient groups stratified by clinical outcome using either ANOVA or Kruskal–Wallis with two-tailed t-test or Mann–Whitney posthoc testing as indicated. Correlation between Ct values of the GeneXpert PA assay and the in-house qPCR and the relation between Ct values of both PCR assays and CFU/ml from the quantitative culture were assessed by linear regression by an F-test as well as by Pearson’s correlation coefficient.

Receiver Operator Characteristic analysis was performed in order to assess the GeneXpert PA assay as a standalone diagnostic for *P. aeruginosa* VAP where the 95% confidence interval was determined using the Wilson/Brown hybrid method.

## Results

We analysed 80 ETA samples utilizing semi-quantitative culture, GeneXpert assay, quantitative culture, enrichment-culture, and O-antigen acetylase gene-based qPCR (overview of results in Additional file 1: Table S1). Sample 70 was *P. aeruginosa*-negative by semi-quantitative culture while *P. aeruginosa*-positive by all the other methods, albeit with low *P. aeruginosa* loads by quantitative culture (2.3 × 10^4^ (1.4% of total growth)) and high Ct values by the in-house qPCR (Ct = 30.1). Sample 70 was thus considered positive which resulted in an extended gold standard with 41 *P. aeruginosa*-positive samples and 39 *P. aeruginosa-*negative samples.

### GeneXpert PA assay is an accurate method for detection of *P. aeruginosa* in ETA samples

With the extended gold standard panel as a comparator, the GeneXpert PA assay showed 97.6% sensitivity (95% CI 87.1–99.9%) and 100% specificity (95% CI 91.0–100%, Table 1). The semi-quantitative culture, quantitative culture, and GeneXpert PA assay showed the highest sensitivity with 97.6%. The sensitivity was not 100% since the GeneXpert PA assay had one negative sample (sample 28) that was also negative by qPCR but confirmed to be positive by semi-quantitative culture and quantitative culture with a load of 40 CFU/ml.

**Table 1:** Analytical performance of the five methods utilized for *P. aeruginosa* detection from ETA samples compared to the extended gold standard

Methods	Extended gold standard^a^		Sensitivity (95% confidence interval)	Specificity (95% confidence interval)
	Positive (n = 41)	Negative (n = 39)		
Quantitative culture				
Positive	40	1	97.6% (87.1–99.9)	97.4% (86.5–99.9)
Negative	1	38		
Enrichment culture (4 samples could not be tested)				
Positive	34	0	89.5% (75.2–97.1)	100% (90.8–100)
Negative	4	38		
In-house qPCR (1 sample could not be tested)				
Positive	38	11	92.7% (80.1–98.5)	71.1% (54.1–84.6)
Negative	3	27		
GeneXpert PA assay				
Positive	40	0	97.6% (87.1–99.9)	100% (91.0–100)
Negative	1	39		
Semi-quantitative culture				
Positive	40	0	97.6% (87.1–99.9)	100% (91.0–100)
Negative	1	39		

^a^*P. aeruginosa* detected by semi-quantitative culture plus one sample that showed *P. aeruginosa* presence by the other four methods but not by semi-quantitative culture

### Semi-quantitative culture has the highest sensitivity and specificity among the culture-based techniques tested

Next, we compared the three culture-based methods, semi-quantitative culture, quantitative culture and enrichment-culture with the extended gold standard results. Semi-quantitative and quantitative culture both showed similar and higher sensitivities (97.6%, 95% CI 87.1–99.9%) than enrichment-culture (89.5%, 95% CI 75.2–97.1%). The specificities of the semi-quantitative culture and the enrichment-culture was 100% (95% CI 91.0–100% and 90.8–100%, respectively) while for the quantitative culture the specificity was 97.4% (95% CI 86.5–99.9%, Table 1). The single sample (sample 70) that was negative for *P. aeruginosa* by semi-quantitative culture had a low PA load. For this sample, Ct values of both PCR methods were above 30 and the load in quantitative culture was 2.3 × 10^4^ CFU/ml, less than 3% compared to the total growth on the blood agar.

Quantitative culture was negative for one sample (sample 41) that was also negative by the enrichment-culture, which indicates a possible heterogeneity of the sample since both the GeneXpert and in-house qPCR detected *P. aeruginosa*. One sample was positive by quantitative culture (sample 63) that was found negative by all other methods where only one *P. aeruginosa* colony was found on blood agar with no growth on the ChromID^®^ *P. aeruginosa* plate. In order to compare bacterial loads, ratio of *P. aeruginosa* in the total bacterial growth (CFU/ml) on quantitative culture gave comparable results to semi-quantitative culture (Additional file 1: Table S1). Enrichment cultures resulted in 34 positive samples out of the in total 38 *P. aeruginosa-*positive samples tested and no positives in the 38 *P. aeruginosa-*negative samples tested. For four samples (7, 36, 39 and 50, marked N/A in Additional file 1: Table S1), the amount was not enough to process with enrichment culture. These were, however, analysed with the other methods. The four false-negatives of the enrichment-based cultures were samples 3, 25, 26 and 31. The latter three had low loads in the semi-quantitative and quantitative culture (< 10^3^ CFU/ml) and the GeneXpert PA assay gave high Ct values (> 30). Sample 3 had a high load in semi-quantitative culture and in quantitative culture above 10^7^ CFU/ml. All of these samples were also negative on the blood agar of the enrichment cultures.

### In-house qPCR showed high sensitivity for *P. aeruginosa* detection in ETA samples

The limit of detection of *P. aeruginosa* in ETA by the in-house qPCR was ≈ 5 × 10^2^ CFU/ml, and the upper limit of quantification was > 10^8^ CFU/ml and is in agreement with the diagnostic thresholds for ETA of ≥ 10^5^ CFU/ml based on quantitative culture [11]. For one sample there was insufficient sample volume for DNA isolation (sample 78). With this qPCR, 49/79 samples tested positive for *P. aeruginosa* and showed a 92.7% (95% CI 80.1–89.5%) sensitivity and a 71.1% (95% CI 54.1–84.6%) specificity with the extended gold standard as reference (Table 1).

**Fig. 2:**
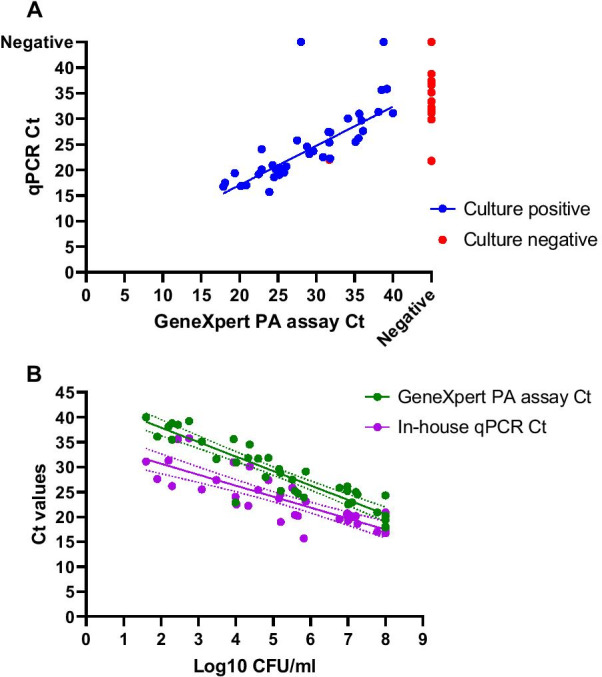
**A** Correlation of Ct values between the GeneXpert PA assay (horizontal-axis) and the in-house qPCR (vertical-axis). Each dot represents a sample and is plotted based on the Ct values from the in-house qPCR (y-axis) and GeneXpert PA assay (x-axis). The line shows the linear regression fit based on the formula: In-house qPCR Ct = 0.7587 × GeneXpert PA Ct + 1.821. Eleven in-house qPCR Cts and three GeneXpert PA assay Cts were not used to calculate the correlation because they were negative in the other assays. **B** Correlation of Ct-values of the GeneXpert PA assay (vertical-axis) and the quantitative culture log10 CFU/ml (horizontal-axis) (green) and the correlation between the in-house RT-qPCR Ct values and the quantitative culture log10 CFU/ml (purple). The lines in the corresponding colors show the linear regression based on the following formulas. For the GeneXpert PA assay: Ct = − 2.900 × log10 CFU/ml + 43.72. The in-house qPCR Ct = − 2.202 × log10 CFU/ml + 35.08

Investigation of the correlation of GeneXpert and in-house qPCR Ct values of all the *P. aeruginosa-*positive samples showed a Pearson correlation of 0.90 (*p* < 0.0001, Fig. 2A). On average for all the samples, the in-house qPCR had a Ct value that was 5.1 Ct lower than the GeneXpert PA assay. This is most likely because the GeneXpert assay detects only intact *P. aeruginosa* cells due to an extra filtration and washing step that removes extracellular *P. aeruginosa* DNA. There was only one sample (sample 18) that showed a lower Ct value (1 Ct) in the GeneXpert PA assay than in the in-house qPCR. Ct values of both the GeneXpert and the in-house qPCR compared to the log10 values of the quantitative culture showed a Pearson correlation of − 0.92 and − 0.83 (*p* < 0.0001), respectively (Fig. 2B).

### Detection of *P. aeruginosa* as a causative agent of VAP

VAP was diagnosed based on a combination of clinical data and the detection of a causative agent as part of routine clinical practice. Based on clinical data in combination with the semi-quantitative cultures performed at the hospital on 13 samples (11 patients) collected within one day of clinical VAP diagnosis (sample no. 4, 5, 7, 10, 13, 14, 17, 18, 20, 21, 30, 31, and 32), nine patients were assigned as having *P. aeruginosa* VAP. All samples from these 9 patients were found positive with all the methods. There were also 10 patients (all with at least one sample within one day of VAP diagnosis,) diagnosed with non-*P. aeruginosa* VAP (sample no. 6, 15, 19, 22, 25, 26, 28, 34, 35, 36, 37, and 39) of which five patients were *P. aeruginosa-*positive although another pathogen was present in higher loads. These results were confirmed in quantitative culture for four of the five patients with loads below 10^5^ CFU/ml. However, ETA from one patient showed an overgrowth of *P. aeruginosa*.

We studied whether the *P. aeruginosa* loads by each of the quantitative assays (except semi-quantitative and enrichment culture that do not have a quantitative output) correlated with VAP diagnosis due to *P. aeruginosa*. Therefore, the samples were split up into four groups: (i) samples from patients with *P. aeruginosa* VAP (PA VAP) (n = 13 samples), (ii) samples from patients with non-*P. aeruginosa* VAP (non-PA VAP) (n = 12), (iii) samples from patients admitted to the ICU with a pneumonia (n = 4), and (iv) samples from patients that did not have any pneumonia signs during their stay or at time of admission (n = 12).

**Fig. 3:**
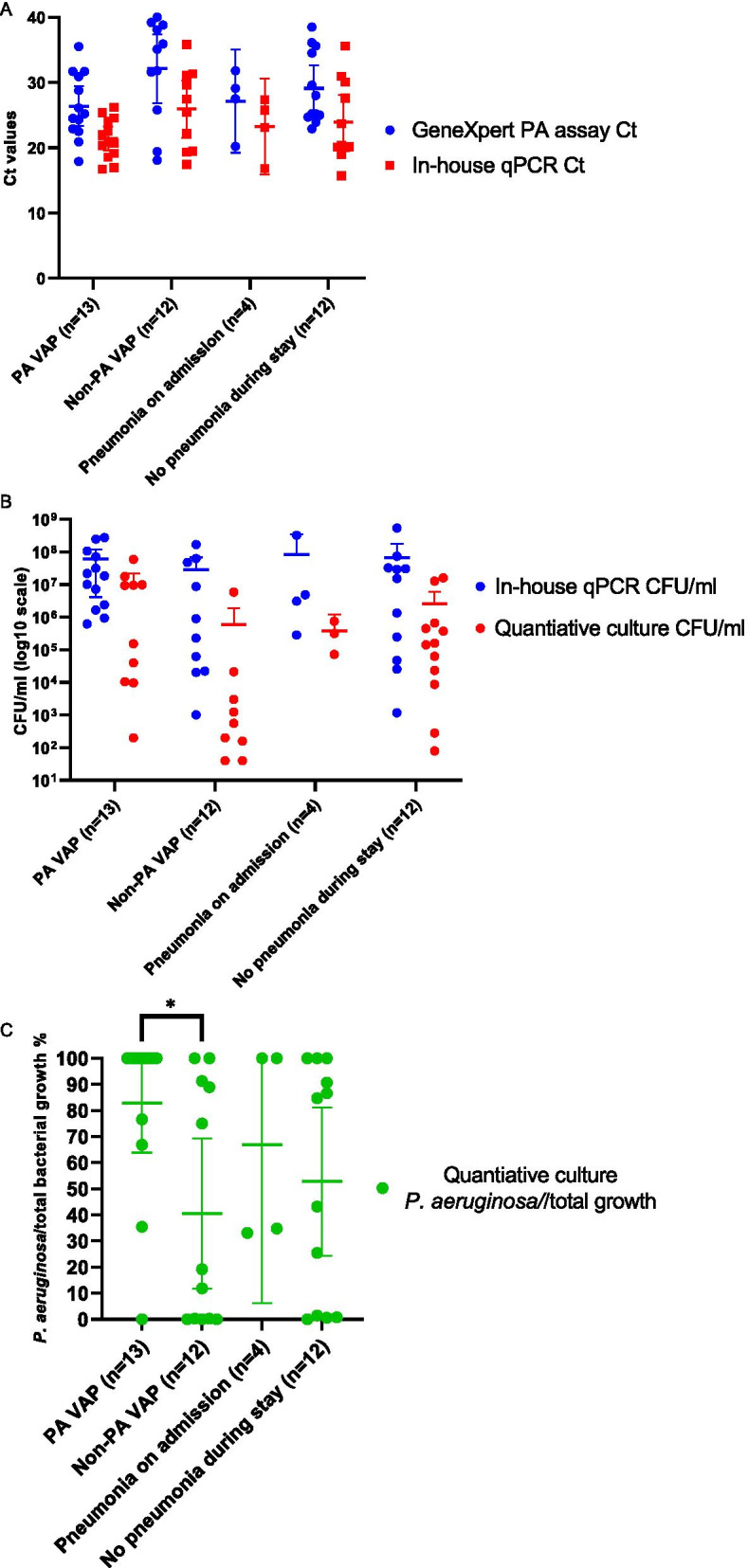
**A** Ct values from GeneXpert PA assay (blue) and in-house qPCR (red) of ETA samples from patients diagnosed with PA VAP, VAP due to other pathogens, pneumonia at admission, or patients without pneumonia. The mean and 95% confidence interval are shown. **B** *P. aeruginosa* loads (CFU/ml) in ETA samples from quantitative culture (red), calculated from the in-house qPCR Ct values using the linear regression equation (blue) of ETA samples from patients diagnosed with PA VAP, VAP due to other pathogens, pneumonia at admission, or patients without pneumonia. The mean and 95% confidence interval are shown. **C** *P. aeruginosa* proportions (% *P. aeruginosa*/total growth on blood agar, in green) calculated from quantitative culture of ETA samples from patients diagnosed with PA VAP, VAP due to other pathogens, pneumonia at admission, or patients without pneumonia. The mean and 95% confidence interval are shown.*Indicates a significant difference (*p* < 0.05) between groups

The loads of *P. aeruginosa* in samples from PA VAP and non-PA VAP patients were compared. The PA VAP group showed lower Ct values than the non-PA VAP group in both qPCR assays (*p* > 0.05 for both GeneXpert PA (PA VAP: 26.4, 23.3–29.4 vs. non-PA VAP: 32.2, 26.9–37.4) and for the in-house qPCR (PA VAP: 21.4, 19.6–23.3 vs. non-PA VAP: 32.2, 26.9–37.4) with a two-tailed T-test, Fig. 3A). No significant differences were found for CFU/ml counts calculated from the in-house qPCR Ct values using the linear regression equation (starting quantity mean (calculated in Biorad CFX maestro 1.1 version 4.1.2433.1219) × 350 × 2) compared to the quantitative culture CFU/ml (Fig. 3B). However, the proportion of *P. aeruginosa* in the total growth in quantitative culture (% *P. aeruginosa*/total growth on blood agar, Fig. 3C) showed a significant difference between the PA VAP and non-PA VAP groups (*p* = 0.014), and a slight difference between the PA VAP and patients that did not develop a pneumonia during their stay (*p* = 0.07). Also no significant differences with any of the three methods were found between *P. aeruginosa* loads in the pre-VAP and the VAP diagnosis samples within the same patient in the four patients where both samples were available.

Finally, we investigated the use of the GeneXpert PA assay as a diagnostic tool for PA VAP using Receiver Operating Characteristic analysis which showed an AUC of 0.69 for samples from patients diagnosed with VAP (95% confidence interval of 0.46–0.93) (Receiver Operating Characteristic curve, Additional file 1: Figure S1, and Fig. 3A). Although a highly sensitive and specific assay, the fact that *P. aeruginosa* detection in samples does not always correlate to PA VAP, as also seen in our study, makes it difficult for any test based purely on *P. aeuginosa* detection to function as a (standalone) diagnostic for PA VAP.

## Discussion

Early and reliable screening for *P. aeruginosa* in the lower respiratory tract may inform targeted and novel preventive strategies like antibody-based therapy (e.g. MEDI3902 clinicaltrials.gov: NCT02696902) [12]. With this in mind, we studied and compared two PCR-based and three culture-based methods for *P. aeruginosa* detection in ETA samples, including semi-quantitative culture routinely used in our hospital. In this study, semi-quantitative culture and the GeneXpert PA assay emerged as the most sensitive and specific methods for detection of *P. aeruginosa* in ETA samples. We recently studied and showed high sensitivity and specificity for GeneXpert MRSA/SA ETA assay which directly detects *S. aureus* from ETA samples compared to the same methods used in this study [13]. The combination of quantitative culture with enrichment culture also resulted in similar sensitivity and specificity as compared to semi-quantitative culture and the GeneXpert PA assay. Nevertheless, with the total hands-on time for preparing the sample of less than 2 min—and with the test completed in less than an hour, the GeneXpert PA assay is a superior method when results need to be received quickly while closely resembling the microbiological diagnosis of semi-quantitative culture. In addition, GeneXpert PA assay is standardized (same method and same equipment used by different laboratories) and therefore should be less affected by cross-laboratory variability than the culture-based methods.

Quantitative culture had a similar sensitivity as the semi-quantitative culture and GeneXpert showing that the pre-treatment with lysomucil was not inhibitory for *P. aeruginosa* recovery. The inhibitory effect of *N*-acetylcysteine on bacterial growth was reported previously [14], however it was not profound in our study as only one sample that had a low load in semi-quantitative culture (non-*N*-acetylcysteine treated) was negative by quantitative culture and enrichment culture (*N*-acetylcysteine treated). Other possible reasons for this discrepancy between the semi-quantitative and quantitative cultures could be because the former was performed directly on samples and the latter post-sample liquefaction. Also, the use of different types of *P. aeruginosa* identification agar plates with both methods might have impacted detection. It has been shown that the ChomID *P. aeruginosa* agar has a slightly lower sensitivity than nalidixic acid-based agar plates [15].

Based on our extended gold standard, the least specific assay was the in-house qPCR where 11 *P. aeruginosa*-negative ETA samples tested positive most likely due to detection of extracellular DNA from the lysed *P. aeruginosa* cells. The discrepancies between the quantifications from culture and the molecular methods could be attributed to different factors. For instance, while culture-negative results could arise due to prior antibiotic use (not recorded in the study) or poor sample handling, the ability to detect bacteria at low concentrations by qPCR, also from extracellular DNA, could (wrongly) classify this test as non-specific in comparison to culture or even to the GeneXpert PA assay. Although a qPCR-based assay, the GeneXpert PA does not pose this problem as it detects only intact bacteria through utilization of an additional filtration and washing step that removes extracellular DNA within the cartridge. This is reflected by the generally higher Ct values of the GeneXpert compared to the in-house qPCR, also making the former assay more comparable with culture-based methods. The combination of only detecting intact bacteria and shorter hands-on time makes the GeneXpert PA assay a valuable and easy tool for *P. aeruginosa* detection in comparison to traditional qPCR assays. It shows the same advantage as a culture-based assay by detecting intact bacteria but has the benefits of being faster with a shorter hands-on time. Thus, it could be an extremely useful assay to screen patients for *P. aeruginosa* colonisation for infection prevention control.

One of the strengths of our study was inclusion of samples from patients that were clinically diagnosed with PA VAP. Of the methods tested in our study, quantitative culture was the most specific in differentiating PA and non-PA VAP. Although in 4 samples from non-PA VAP, the *P. aeruginosa* load was above the PA VAP average (84% CI 63.8–102%) underscoring the potential pitfall of utilising *P. aeruginosa* loads to be indicative of VAP.

The fact that quantitative culture which calculates the ratio of *P. aeruginosa*/total growth is still the most accurate assay for detection of PA VAP might be related to prior received empirical treatment with (broad-spectrum) antibiotics that decrease the correlation with PCR-based Ct values (Fig. 2A). Samples from both PA and non-PA VAP showed overlaps in Ct values for both the in-house qPCR and the GeneXpert PA assay. Although 61.5% and 53.8% of PA VAP diagnosed patients showed Ct values of < 26.3 and < 21.4 with the GeneXpert PA assay and in-house qPCR respectively, the samples from non-PA VAP also showed Ct values below those ranges however the percentages were lower (25% for both the GeneXpert PA assay and the in-house qPCR). The receiver operating characteristic curve (Additional file 1: Figure S1) shows that the GeneXpert is useful yet would result in over-diagnosis of PA VAP. Further, the currently used method, semi-quantitative culture, has the advantage of detecting other co-existing pathogens in ETA samples. As seen in some of the VAP patients included in our study, *P. aeruginosa* was detected however VAP was attributed to another pathogen that was present at an even higher abundance.

Although all methods showed good sensitivity, none was 100% sensitive compared to the extended gold standard. In the enrichment cultures and qPCR, four and three samples, respectively, were negative while they were positive by the extended gold standard method. The BHI enrichment culture was performed using leftover sample after quantitative culture and in-house qPCR which might have resulted in too small of an inoculum, as indicated by the negative blood agar plates after enrichment. Two false negative samples in the qPCR had low loads as confirmed by semi-quantitative and quantitative culture and one was also negative with the GeneXpert PA assay while the other had high Ct values. The third sample had a moderate load in semi-quantitative culture which was reflected in both the GeneXpert PA assay and the quantitative culture. A possible explanation for the negative result might be a low homogeneity of the sample. The results of the enrichment cultures are counter intuitive since a high sensitivity is to be expected and a lower specificity. Sample 31 might be affected by the heterogeneity of the sample since both the quantitative and enrichment culture were falsely negative. The other assays showed that not only *P. aeruginosa* was present and it might have been outcompeted by other bacteria in the BHI broth.

However, the GeneXpert PA assay provides a fast and accurate method highlighting its potential as a screening assay to detect *P. aeruginosa* in mechanically ventilated patients to take forward preventive strategies that require early and rapid detection of *P. aeruginosa* colonization.

## Conclusion

*P. aeruginosa* is a common cause of VAP, a frequent nosocomial infection. Rapid and accurate detection of lower respiratory tract colonization and/or infection with *P. aeruginosa* may inform targeted preventive and therapeutic strategies. The GeneXpert PA assay is a recently introduced molecular test directly detecting *P. aeruginosa* in ETA samples with an average turnaround time of 60 min. We show here for the first time that the GeneXpert PA assay is a rapid and sensitive method for direct detection of *P. aeruginosa* in the ETA samples with 97.6% sensitivity and 100% specificity in comparison to the extended gold standard method.

## Supplementary Information

**Additional file 1.** Results from the five assays evaluated in this study.

## Data Availability

Please contact the author for data requests.

## Abbreviations

ETA: Endotracheal aspirates
VAP: Ventilator-associated pneumonia
ICU: Intensive care unit
qPCR: Quantitative TaqMan real-time PCR
Ct: Cyclic threshold
CFU: Colony-forming units
BHI: Brain heart infusion
