# Supplementary material for: Evaluation of GeneXpert PA assay compared to genomic and (semi-)quantitative culture methods for direct detection of Pseudomonas aeruginosa in endotracheal aspirates
Source: Antimicrob Resist Infect Control. 2021 Jul 23;10:110. doi: 10.1186/s13756-021-00978-9 (PMC8300976; doi:10.1186/s13756-021-00978-9)
Supplement: Supplementary file 1 — Additional file 1. Results from the five assays evaluated in this study. [file 13756_2021_978_MOESM1_ESM.docx]

## Supplementary table

**Supplementary table 1**: Overview of the results obtained by analyzing 80 ETAs using semi-quantitative culture, the GeneXpert assay, quantitative culture, enrichment-based culture, and in-house qPCR. +/-: Sample detected positive or negative for *P. aeruginosa*. Several samples were not available (N/A) for certain assays. Heavy, moderate and light growth in the semi-quantitative culture were assigned based on the quadrants of growth (quadrants 4, 2-3 and 1, respectively).

| **Sample #** | **Extended gold standard** | **Semi-quantitative culture** | **GeneXpert PA assay** | | **Quantitative culture** | | | **Enrichment** | **qPCR** | |
| --- | --- | --- | --- | --- | --- | --- | --- | --- | --- | --- |
|  |  |  | ***P. aeruginosa* detected** | **Ct value** | ***P. aeruginosa* detected** | **CFU/ml** | ***P. aeruginosa* CFU/ml/total growth CFU/ml (%)** | ***P. aeruginosa* detected** | ***P. aeruginosa* detected** | **Ct value** |
| 1 | + | heavy | + | 20.2 | + | too many | 100 | + | + | 16.8 |
| 2 | + | heavy | + | 22.9 | + | 1.28E+07 | 100 | + | + | 20.1 |
| 3 | + | heavy | + | 25.0 | + | 1.63E+07 | 25.5 | - | + | 20.2 |
| 4 | + | heavy | + | 31.7 | + | 4.00E+04 | 100 | + | + | 25.4 |
| 5 | + | heavy | + | 22.5 | + | 1.00E+07 | 100 | + | + | 19.2 |
| 6 | + | heavy | + | 18.1 | + | too many | 91.3 | + | + | 17.5 |
| 7 | + | heavy | + | 17.9 | + | too many | 100 | N/A | + | 16.7 |
| 8 | + | heavy | + | 24.7 | + | 4.52E+05 | 43.2 | + | + | 20.2 |
| 9 | + | heavy | + | 23.9 | + | 6.67E+05 | 86.6 | + | + | 15.7 |
| 10 | + | heavy | + | 26.1 | + | 9.64E+06 | 66.9 | + | + | 20.7 |
| 11 | + | moderate | + | 25.2 | + | 1.60E+05 | 84.7 | + | + | 19.0 |
| 12 | + | moderate | + | 28.0 | + | 6.32E+04 | 90.7 | + | - | >40 |
| 13 | + | moderate | + | 24.5 | + | 1.78E+07 | 100 | + | + | 18.6 |
| 14 | + | moderate | + | 25.2 | + | 9.49E+06 | 100 | + | + | 20.4 |
| 15 | + | moderate | + | 19.4 | + | too many | 100 | + | + | 19.4 |
| 16 | + | moderate | + | 25.2 | + | 3.80E+05 | 100 | + | + | 20.4 |
| 17 | + | moderate | + | 20.9 | + | 5.96E+07 | 100 | + | + | 17.0 |
| 18 | + | moderate | + | 22.9 | + | 9.76E+03 | 76.6 | + | + | 24.0 |
| 19 | + | moderate | + | 25.8 | + | 5.91E+06 | 100 | + | + | 19.5 |
| 20 | + | moderate | + | 24.3 | + | too many | 100 | + | + | 20.9 |
| 21 | + | low | + | 28.8 | + | 1.56E+05 | 100 | + | + | 24.5 |
| 22 | + | low | + | 31.6 | + | 3.04E+03 | 88.9 | + | + | 27.4 |
| 23 | + | low | + | 29.6 | + | 1.43E+05 | 0.627 | + | + | 23.7 |
| 24 | + | low | + | 36.1 | + | 8.00E+01 | 100 | + | + | 27.6 |
| 25 | + | low | + | 40.0 | + | 4.00E+01 | 0.257 | - | + | 31.1 |
| 26 | + | low | + | 35.1 | + | 1.24E+03 | 75.0 | - | + | 25.5 |
| 27 | + | low | + | 38.5 | + | 2.80E+02 | 0.0002 | + | + | 35.6 |
| 28 | + | low | - | >45 | + | 4.00E+01 | 0.00 | + | - | >40 |
| 29 | + | low | + | 31.8 | + | 7.34E+04 | 33.1 | + | + | 27.4 |
| 30 | + | low | + | 35.5 | + | 2.00E+02 | 100 | + | + | 26.2 |
| 31 | + | low | + | 31.7 | - | 0.00E+00 | 0.00 | - | + | 22.0 |
| 32 | + | low | + | 30.9 | + | 1.05E+04 | 35.5 | + | + | 22.5 |
| 33 | + | low | + | 35.6 | + | 8.72E+03 | 0.755 | + | + | 31.0 |
| 34 | + | low | + | 39.2 | + | 5.60E+02 | 0.0091 | + | + | 35.8 |
| 35 | + | low | + | 38.8 | + | 2.00E+02 | 0.0491 | + | - | >40 |
| 36 | + | low | + | 35.9 | + | 0.00E+00 | 11.9 | N/A | + | 29.7 |
| 37 | + | low | + | 38.1 | + | 1.60E+02 | 0.260 | + | + | 31.3 |
| 38 | + | low | + | 29.1 | + | 7.48E+05 | 100 | + | + | 23.1 |
| 39 | + | low | + | 31.8 | + | 2.14E+04 | 19.2 | N/A | + | 22.2 |
| 40 | + | low | + | 27.5 | + | 3.20E+05 | 34.8 | + | + | 25.8 |
| 41 | - | negative | - | >45 | - | 0.00E+00 | 0.00 | - | - | >40 |
| 42 | - | negative | - | >45 | - | 0.00E+00 | 0.00 | - | - | >40 |
| 43 | - | negative | - | >45 | - | 0.00E+00 | 0.00 | - | - | >40 |
| 44 | - | negative | - | >45 | - | 0.00E+00 | 0.00 | - | + | 37.2 |
| 45 | - | negative | - | >45 | - | 0.00E+00 | 0.00 | - | + | 29.9 |
| 46 | - | negative | - | >45 | - | 0.00E+00 | 0.00 | - | - | >40 |
| 47 | - | negative | - | >45 | - | 0.00E+00 | 0.00 | - | + | 37.4 |
| 48 | - | negative | - | >45 | - | 0.00E+00 | 0.00 | - | - | >40 |
| 49 | - | negative | - | >45 | - | 0.00E+00 | 0.00 | - | - | >40 |
| 50 | - | negative | - | >45 | - | 0.00E+00 | 0.00 | N/A | - | >40 |
| 51 | - | negative | - | >45 | - | 0.00E+00 | 0.00 | - | + | 35.1 |
| 52 | - | negative | - | >45 | - | 0.00E+00 | 0.00 | - | + | 31.3 |
| 53 | - | negative | - | >45 | - | 0.00E+00 | 0.00 | - | + | 32.2 |
| 54 | - | negative | - | >45 | - | 0.00E+00 | 0.00 | - | - | >40 |
| 55 | - | negative | - | >45 | - | 0.00E+00 | 0.00 | - | - | >40 |
| 56 | - | negative | - | >45 | - | 0.00E+00 | 0.00 | - | - | >40 |
| 57 | - | negative | - | >45 | - | 0.00E+00 | 0.00 | - | - | >40 |
| 58 | - | negative | - | >45 | - | 0.00E+00 | 0.00 | - | - | >40 |
| 59 | - | negative | - | >45 | - | 0.00E+00 | 0.00 | - | - | >40 |
| 60 | - | negative | - | >45 | - | 0.00E+00 | 0.00 | - | - | >40 |
| 61 | - | negative | - | >45 | - | 0.00E+00 | 0.00 | - | - | >40 |
| 62 | - | negative | - | >45 | - | 0.00E+00 | 0.00 | - | - | >40 |
| 63 | - | negative | - | >45 | + | 0.00E+00 | 0.00 | - | - | >40 |
| 64 | - | negative | - | >45 | - | 0.00E+00 | 0.00 | - | - | >40 |
| 65 | - | negative | - | >45 | - | 0.00E+00 | 0.00 | - | - | >40 |
| 66 | - | negative | - | >45 | - | 0.00E+00 | 0.00 | - | - | >40 |
| 67 | - | negative | - | >45 | - | 0.00E+00 | 0.00 | - | - | >40 |
| 68 | - | negative | - | >45 | - | 0.00E+00 | 0.00 | - | - | >40 |
| 69 | - | negative | - | >45 | - | 0.00E+00 | 0.00 | - | + | 21.8 |
| 70 | +* | negative | + | 34.5 | + | 2.34E+04 | 1.49 | + | + | 30.1 |
| 71 | - | negative | - | >45 | - | 0.00E+00 | 0.00 | - | + | 36.6 |
| 72 | - | negative | - | >45 | - | 0.00E+00 | 0.00 | - | - | >40 |
| 73 | - | negative | - | >45 | - | 0.00E+00 | 0.00 | - | - | >40 |
| 74 | - | negative | - | >45 | - | 0.00E+00 | 0.00 | - | - | >40 |
| 75 | - | negative | - | >45 | - | 0.00E+00 | 0.00 | - | - | 0 |
| 76 | - | negative | - | >45 | - | 0.00E+00 | 0.00 | - | + | 31.2 |
| 77 | - | negative | - | >45 | - | 0.00E+00 | 0.00 | - | + | 33.4 |
| 78 | - | negative | - | >45 | - | 0.00E+00 | 0.00 | - | N/A | N/A |
| 79 | - | negative | - | >45 | - | 0.00E+00 | 0.00 | - | + | 38.8 |
| 80 | - | negative | - | >45 | - | 0.00E+00 | 0.00 | - | - | 0 |

*Sample 70 was negative for P. aeruginosa by semi-quantitative culture, however was detected positive by the other four methods used here. Thus, this was included as a P. aeruginosa positive samples in the extended gold standard used for calculating sensitivity and specificity of each test.

## Supplementary figure

**Supplementary figure 1**: Receiver Operating Characteristic curves for the GeneXpert PA assay for *P. aeruginosa* VAP diagnosis. Curve show the resulting sensitivity and specificity for all included patient data in the study (blue) including negative GeneXpert PA assay results (which were set at Ct of 40 for this analysis) and that for PA VAP patient samples only (red). The area under the curve is 0.87 (95% confidence interval: 0.79-0.95) and 0.69 (95% confidence interval of 0.46-0.93) for all patients and the *P. aeruginosa*-positive patients only, respectively.
